# HMGA1 drives stem cell, inflammatory pathway, and cell cycle progression genes during lymphoid tumorigenesis

**DOI:** 10.1186/1471-2164-12-549

**Published:** 2011-11-04

**Authors:** Andrew Schuldenfrei, Amy Belton, Jeanne Kowalski, C Conover Talbot, Francescopaolo Di Cello, Weijie Poh, Hua-Ling Tsai, Sandeep N Shah, Tait H Huso, David L Huso, Linda MS Resar

**Affiliations:** 1 Department of Medicine, Division of Hematology, Johns Hopkins University School of Medicine, 720 Rutland Avenue, Baltimore, MD, 21205, USA; 2 Department of Oncology, Division of Oncology Biostatistics, Sidney Kimmel Comprehensive Cancer Center at Johns Hopkins, 550 North Broadway, Baltimore, MD 21205, USA; 3 Institute for Basic Biomedical Sciences, Johns Hopkins University School of Medicine, 733 North Broadway, Baltimore, MD, 21205, USA; 4 Pathobiology Graduate Program, Johns Hopkins University School of Medicine, 720 Rutland Avenue, Baltimore, MD, 21205, USA; 5 Department of Molecular and Comparative Pathobiology, Johns Hopkins University School of Medicine, 733 North Broadway, Baltimore, MD, 21205, USA; 6 Department of Oncology, Johns Hopkins University School of Medicine, 720 Rutland Avenue, Baltimore, MD 21205, USA; 7 Department of Pediatrics, Johns Hopkins University School of Medicine, 720 Rutland Avenue, Baltimore, Maryland 21205

## Abstract

**Background:**

Although the *high mobility group A1 *(*HMGA1*) gene is widely overexpressed in diverse cancers and portends a poor prognosis in some tumors, the molecular mechanisms that mediate its role in transformation have remained elusive. *HMGA1 *functions as a potent oncogene in cultured cells and induces aggressive lymphoid tumors in transgenic mice. Because HMGA1 chromatin remodeling proteins regulate transcription, *HMGA1 *is thought to drive malignant transformation by modulating expression of specific genes. Genome-wide studies to define HMGA1 transcriptional networks during tumorigenesis, however, are lacking. To define the HMGA1 transcriptome, we analyzed gene expression profiles in lymphoid cells from *HMGA1a *transgenic mice at different stages in tumorigenesis.

**Results:**

RNA from lymphoid samples at 2 months (before tumors develop) and 12 months (after tumors are well-established) was screened for differential expression of > 20,000 unique genes by microarray analysis (Affymetrix) using a parametric and nonparametric approach. Differential expression was confirmed by quantitative RT-PCR in a subset of genes. Differentially expressed genes were analyzed for cellular pathways and functions using Ingenuity Pathway Analysis. Early in tumorigenesis, HMGA1 induced inflammatory pathways with NFkappaB identified as a major node. In established tumors, HMGA1 induced pathways involved in cell cycle progression, cell-mediated immune response, and cancer. At both stages in tumorigenesis, HMGA1 induced pathways involved in cellular development, hematopoiesis, and hematologic development. Gene set enrichment analysis showed that stem cell and immature T cell genes are enriched in the established tumors. To determine if these results are relevant to human tumors, we knocked-down HMGA1 in human T-cell leukemia cells and identified a subset of genes dysregulated in both the transgenic and human lymphoid tumors.

**Conclusions:**

We found that *HMGA1 *induces inflammatory pathways early in lymphoid tumorigenesis and pathways involved in stem cells, cell cycle progression, and cancer in established tumors. *HMGA1 *also dyregulates genes and pathways involved in stem cells, cellular development and hematopoiesis at both early and late stages of tumorigenesis. These results provide insight into *HMGA1 *function during tumor development and point to cellular pathways that could serve as therapeutic targets in lymphoid and other human cancers with aberrant *HMGA1 *expression.

## Background

The *high mobility group A1 *gene (*HMGA1*) is highly expressed in virtually all poorly differentiated or refractory cancers studied to date, although its role in this setting is poorly understood [1-23]. *HMGA1 *(formerly *HMG-I/Y*) encodes the HMGA1a and HMGA1b (formerly HMG-I and HMG-Y) chromatin remodeling proteins, which function in modulating gene expression [16-19]. These protein isoforms result from alternatively spliced mRNA and differ by 11 internal amino acids present in the HMGA1a isoform [23-25]. HMGA1 proteins are members of the superfamily of HMG proteins named for their rapid electrophoretic mobility in polyacrylamide gels (thus high mobility group). The HMGA family is distinguished by AT-hook DNA binding domains that mediate binding to AT-rich regions in the minor groove of chromatin and includes HMGA1 and HMGA2 proteins [16,26-33]. After binding to DNA, HMGA1 proteins recruit additional transcription factors and histone modifying proteins to alter chromatin structure and form a higher order transcriptional complex or enhanceosome [16]. In concert with these factors, HMGA1 proteins modulate gene expression. Although its role in transcription is well-established, the downstream transcriptional targets regulated by HMGA1 proteins are only beginning to emerge.

HMGA1 proteins were first linked to cancer over 25 years ago when they were discovered as abundant, nonhistone chromatin binding proteins in human HeLa cervical cancer cells [27]. Subsequent studies showed highly expression levels in poorly differentiated cancers, refractory hematologic malignancies, and the developing embryo, with low or undetectable levels in most adult, differentiated tissues [1-3,5-7,9-27,32-39]. HMGA1 proteins induce oncogenic phenotypes in cultured cells, including enhanced proliferation, anchorage-independent cell growth, migration and invasive properties, and xenograft tumorigenesis when implanted into immunosuppressed mice [2-19]. HMGA1 also promotes metastatic progression and an epithelial-to-mesenchymal transition in a breast cancer model [18]. Transgenic mice misexpressing HMGA1 develop aggressive tumors involving all three germ layers [6,11,34]. Conversely, inhibiting *HMGA1 *expression or function in experimental models blocks transformation phenotypes such as proliferation, anchorage-independent cell growth, tumorigenesis, and metastatic progression [2,5,10-13,15,18,35,36]. Further, *HMGA1 *gene expression and protein levels correlate with adverse clinical outcomes [11,14-16,37-39]. For example, *HMGA1 *gene expression was identified as a marker of poor outcome in pediatric brain tumors [37]. High HMGA1 protein levels correlate with poor differentiation in pancreatic and breast cancers [14,38] and decreased survival in pancreatic and lung cancers [14,39] by immunohistochemical analysis in primary tumors. More recently, HMGA1 was identified as a key transcription factor enriched in embryonic stem cells and poorly differentiated cancers of the breast, bladder, and brain [1]. Expression of HMGA1 and 8 other transcriptional regulators predicted poor survival in these cancers. HMGA1 is also enriched in hematopoietic stem cells [20,21,40]. Together, these studies implicate HMGA1 as a key regulator in tumor progression, poor differentiation, and refractory disease.

To better elucidate the role of HMGA1 in tumor progression, we used genome-wide expression profile analysis in our *HMGA1a *(herein referred to as *HMGA1*) transgenic mouse model for lymphoid malignancy. These mice succumb to acute lymphoid leukemia by 8-13 months of age with complete penetrance. Here, we identified genes dysregulated by HMGA1 early in tumorigenesis and in well-established tumors. Our studies reveal distinct pathways activated by HMGA1 at different stages in tumor development. Although further studies are needed, our results uncover cellular pathways that could serve as potential therapeutic targets in lymphoid and other tumors with aberrant expression of *HMGA1*.

## Methods

### HMGA1 transgenic mice lymphoid samples

The *HMGA1 *mice have been previously reported [6,11]. Briefly, the murine *Hmga1a *cDNA is driven by the H-2K promoter and immunoglobulin μ enhancer, which is expressed in B and T cells. All mice develop lymphoid malignancy by 5-10 months and die from their disease by 8-13 months [6]. The female mice also develop uterine sarcomas [11]. RNA was isolated from the splenocytes at different stages of developing tumorigenesis, including 2 months (before the onset of tumors), and at 12 months (when tumors are well established). Splenic RNA was chosen because these organs are abundant sources of lymphoid cells and they become uniformly infiltrated with leukemic cells in all transgenics by 9-12 months. Splenocytes from nontransgenic littermates were harvested at the same time points as controls.

### Isolation of total RNA

Total RNA was isolated from control and transgenic mouse spleens that were harvested at necropsy and immediately suspended in RNAlater (Qiagen) to prevent RNA degradation. From each spleen sample, approximately 15-30 mg of spleen tissue was homogenized in RLT buffer (Qiagen) with the Power Gen 700 homogenizer (Fisher Scientific). Total RNA was purified using the RNeasy Mini kit with DNase treatment (Qiagen) to eliminate genomic DNA according to the manufacturer's instructions. RNA was measured with the NanoDrop 1000 (Thermo Scientific) and purity was assessed with a 2100 Bioanalyzer (Agilent technologies). Total RNA was isolated from 2 spleens from each age group and pooled as a single sample to a final concentration of 600 ng/μl for microarray analysis. Two independent, pooled samples were prepared at each time point from transgenic and control mice.

### Microarray analysis

The microarray analysis was performed at the Johns Hopkins University School of Medicine Microarray Core facility as previously described [41-44]. Briefly, mRNA was converted into double stranded cDNA using a T7-oligo (dT) promoter primer sequence and purified for use as a template for *in vitro *transcription. Transcription reactions were performed with T7 polymerase and biotinylated nucleotide analog/ribonucleotide mix for cRNA amplification. The biotinylated cRNA was prepared in the hybridization mix with oligonucleotide B2 and four control bacterial and phage cDNA. Labeled cRNA was hybridized to the Mouse 430 2.0 GeneChip array (Affymetrix) containing 45,000 probesets corresponding to 21,814 unique genes according to the manufacturer's instructions. Experiments were performed with two independent samples at each time point from the transgenic and control mice. All reported microarray data are MIAME compliant and stored in the Gene Expression Ominbus (GEO). The data can be accessed at GEO under accession number GSE22922.

### Data analysis

We used both a parametric statistical approach (Partek Genomics Suite) and a nonparametric approach [41] to generate a list of differentially expressed genes. The overlapping, differentially expressed genes from both approaches were then analyzed for specific cellular pathways and gene set enrichment patterns.

All data were analyzed using the Partek Genomics Suite v6.4 (Partek Inc., St. Louis, MO, USA). The data was adjusted for GC content, normalized with quantile normalization and mean probe summarization by Robust Multichip Average (RMA) [43,44], and expression fold change values were determined. Significantly dysregulated probesets were identified using Spotfire DecisionSite 9 (TIBCO, Palo Alto, CA). Probes were selected if they fulfilled two criteria: 1) change in expression of ≥ 1.3, and, 2) a paired p-value indicating significance. Paired p-values were obtained through Partek's paired sample t-test to reduce batch effects by comparing samples prepared at the same time. We generated lists of probesets with a p ≤ 0.05 in addition to a more selective list with a p ≤ 0.01. Further pathway and gene set enrichment analysis were conducted on the dataset with p ≤ 0.05.

As an alternative method to the parametric approach, a nonparametric approach (Correlative Analysis of Microarray or CAM) was implemented as previously described [41] and the results from the two statistical approaches were compared for concordance. Briefly, the CAM approach was developed for high-dimensional data analyses from microarray gene expression studies with limited sample numbers per comparison group. Because we noted a batch effect with different scale and expression distributions in the replicate experiments, we separately analyzed the data generated from the comparison of the transgenic and control samples from each experiment and compared the differentially regulated gene sets between replicates for concordance to identify a common gene set from each time point.

The list of probesets was filtered for duplicates. All probesets that were not annotated or ambiguously annotated were checked by BLAST comparison of their sequences against the online NCBI37/mm9 mouse genome sequence in the UCSC Genome Browser (Genome Bioinformatics Group of UC Santa Cruz, CA, USA) to ensure accuracy.

### Pathway analysis

To elucidate cellular pathways regulated by HMGA1 early in tumorigenesis, Ingenuity Pathway Analysis (IPA, Ingenuity^® ^Systems, http://www.ingenuity.com) of selected genes from our microarray data was used. The IPA score was generated for each comparison and indicates the likelihood that the focus genes present in the network could have been obtained solely by chance. A score ≥ 3 was considered significant because it represents a 1/1,000 chance that the network contains the specific focus genes by random chance alone [45]. For each network, pathways or functions from the following categories were generated: 1.) disease or disorder-related functions, 2.) molecular and cellular functions, and, 3.) physiological system development and function. The pathway with the most significant p value was determined from each analysis and the top biologic function was defined as the pathway or function from all categories with the most significant p value.

### Gene set enrichment analysis

Gene set enrichment analysis was performed as previously described using GSEA v2.0 software to identify published gene expression profiles that share genes with the profiles identified in this study (http://www.broad.mit.edu/gsea) [46,47]. The p value indicates the significance of the overlap.

### Quantitative real-time PCR validation (qRT-PCR)

The change in expression of candidate genes that were differentially regulated by *HMGA1 *by microarray approaches was confirmed by qRT-PCR. qRT-PCR was performed as we described [6,11], except for the following modifications. Total RNA was isolated from mouse spleens using the RNeasy kit (Qiagen) as recommended by the manufacturer. cDNA was generated by reverse transcription of total RNA (500 ng; ABI High Capacity cDNA Reverse Transcription kit) and subsequently analyzed by qRT-PCR using the ABI SYBR Green assay kit. Reaction conditions were 2 min at 50°C, 10 min at 95°C, followed by 40 cycles of 15 s at 95°C and 60 s at 60°C. qRT-PCR results were analyzed by normalizing expression to the internal control glyceraldehyde-3-phosphate dehydrogenase (GAPDH). Primers for all genes excluding *HMGA1 *and *GAPDH *were designed using the IDT Primer Quest software and the primer sequences are listed (Table 1). The *HMGA1 *and *GAPDH *primers have been previously reported [11].

**Table 1:** Primers for validation of differentially expressed murine and human genes

Gene	Forward Primer	Reverser Primer	RefSeq ID
***Mouse Primers***			
***Bub1b***	TAGTGGCTTTCGGACTGCACAGAT	AGACATGGAGGTGCTCCTTGAACA	[NCBI:NM_009773.3]
***Cd8b1***	CTGCTTTGAACTGCTGCAAGCTCT	TGGGAGTTCTTGGTTCTTCAGCCA	[NCBI:NM_009858.2]
***Cxcr3***	AACTCAGCCATCCCTGTGTGAGAA	ATGGGCACATTCAGTGCTGACAAC	[NCBI:NM_009910.2]
***Eomes***	AGGTCGTTCAAGGTGCTGGATTGA	TAATAGCACCGGGCACTCGTTCTT	[NCBI:NM001 164789.1]
***Foxp1***	ACTCTGTGCATTGGATGGACCTGT	AAGCTGCAGTTCAAAGTCTGCTGC	[NCBI:NM001197321.1]
***Gzmm***	ACCTTCTACATCCGGGAAGCCATT	GTGGTTTGACATTCTTGCTGGGCT	[NCBI:NM_008504.2]
***Il18r1***	ATCCTGAAGGATGCCGAGTTTGGA	TGGTGATGTTGTACCGTGTCCCAT	[NCBI:NM001161842.1]
***Il2rb***	TTTCTGGCTTCTTCTCCTGCGTCT	AAGGATCTGGGATGTGGCACTTGA	[NCBI:NM008368.4]
***Human Primers***			
***BUB1B***	TGGGATGGGTCCTTCTGGAAACTT	CACTGTGGCCTCATCATTGGCATT	[NCBI:NM001211.5]
***CD8B1***	ACCTCACAGAAGCTGCTTAACCCA	TGAGCGAGGGAGGAATCTGGTAAA	[NCBI:NM001178100.1]
***CXCR3***	ACATAGTTCATGCCACCCAGCTCT	TGGGAAGTTGTATTGGCAGTGGGT	[NCBI:NM001142797.1]
***EOMES***	CAAATTCCACCGCCACCAAACTGA	TTGTAGTGGGCAGTGGGATTGAGT	[NCBI:NM_005442.2]
***FOXP1***	AAACATTTCGGCAATGGTGAGGGC	TGCATAATGCCACAGGACTGCAAC	[NCBI:NM_001012505.1]
***GZMM***	GTCTGCACTGACATCTTCAAGCCT	ATTTATTGGTCCCTCCCTGTCCCT	[NCBI:NM005317.2]
***IL18R1***	CTCCAGAAGGCAAATGGCATGCTT	ATTCCTCTTAAGACGTGGCCT	[NCBI:NM003855.2]
***IL2RB***	TCCCAAGCCTCCCACTACTTTGAA	TGACCCGCACCTGAAACTCATACT	[NCBI:NM_000878.2]

### Knock-down of HMGA1 in human leukemia cells

*HMGA1 *expression was knocked-down using siRNA to *HMGA1 *(Dharmacon) as we previously described [11], but with the following modifications. Jurkat cells (5 × 10^6^), a human T-cell acute lymphoblastic cell line, were transfected with the *HMGA1 *siRNA (6 ug) by nucleofection with the Amaxa nucleofector kit V (Lonza, Inc.) according to the manufacturer's instructions. Control Jurkat cells (5 × 10^6^) were treated with the siGenome control non-targeting control siRNA (6 ug; Dharmacon). Cells were subsequently seeded onto six well plates and collected at 24, 48, and 72 hours for protein and mRNA. To document *HMGA1 *knock-down, both qRT-PCR for mRNA and Western analysis for protein were performed as previously described [11].

## Results

### The genes differentially regulated by HMGA1 early and late in tumorigenesis

#### Nonparametric CAM approach

Early in tumorigenesis (at 2 months of age), 46 probes representing 40 unique genes were significantly up-regulated and 86 probes or 73 unique genes were significantly down-regulated in the *HMGA1 *transgenics compared to controls in replicate samples by the nonparametric correlative analysis of microarrays (CAM) approach [41]. In established tumors at 12 months, 289 probes or 260 unique genes were significantly up-regulated and 246 probes or 165 unique genes were significantly down-regulated in the transgenics compared to controls in replicate samples (Table 2).

**Table 2:** List of probe hits and corresponding genes at each time point

Up-regulated				
**Age/Tumor status**	**Method**	**Probe Hits**	**Unique Genes**	**Overlap**

**2 months/Early in tumorigenesis**	CAM	46	40	

	Partek (p ≤ 0.05)	19	18	11

	Partek (p ≤ 0.01)	7	7	4

**12 months/Established tumors**	CAM	296	260	

	Partek (p ≤ 0.05)	303	283	71

	Partek (p ≤ 0.01)	64	63	16

**Down-regulated**				

**Age/Tumor status**	**Method**	**Probe Hits**	**Unique Genes**	**Overlap**

**2 months/Early in tumorigenesis**	CAM	86	73	

	Partek (p ≤ 0.05)	18	18	10

	Partek (p ≤ 0.01)	7	7	5

**12 months/Established tumors**	CAM	246	165	

	Partek (p ≤ 0.05)	470	432	46

	Partek (p ≤ 0.01)	92	89	13

The number of differentially expressed probes hits and corresponding unique genes identified by a parametric (Partek) approach or nonparametric (CAM) approach are shown The overlap of genes identified by both approaches is also shown for p ≤ 0.05 or ≤ 0.01.

#### Parametric Partek analysis

By the parametric approach [42], 36 genes were differentially regulated by ≥ 1.3-fold with 18 genes up-regulated and 18 down-regulated (p ≤ 0.05) early in tumorigenesis. With a more stringent p value of ≤ 0.01, 14 genes were differentially regulated by ≥ 1.3-fold with 7 genes up-regulated and 7 down-regulated. In the established tumors at 12 months, 715 genes were differentially regulated (≥ 1.3 fold) with 283 genes up-regulated and 432 down-regulated (p ≤ 0.05). With the p value of ≤ 0.01, 152 genes were differentially regulated by (≥ 1.3-fold) with 63 genes up-regulated and 89 genes down-regulated (Table 2). There was a very high correlation between the differentially regulated genes identified in experiment 1 compared to experiment 2, with an R > 0.99 at both the early and late time points.

### The cellular pathways dysregulated by HMGA1 early in tumorigenesis

#### Nonparametric analysis

**Figure 1:**
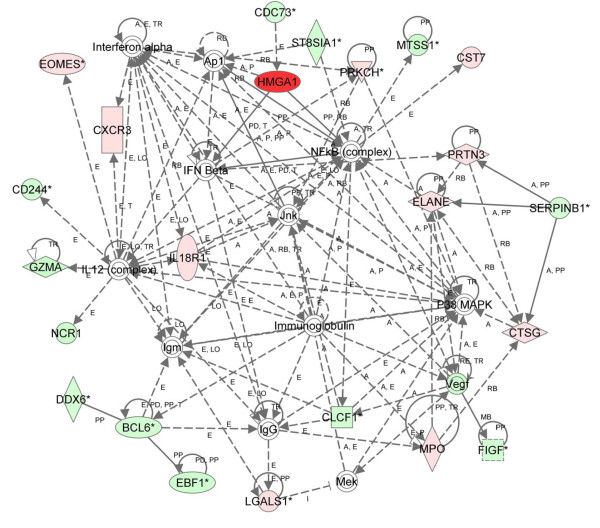
**Network of differentially expressed genes in *HMGA1 *transgenics at 2 months (nonparametric CAM approach)**. IPA was performed on microarray data comparing transgenic to control lymphoid cells from mice. Using 113 differentially expressed genes as the focus gene set identified by nonparametric analysis, the highest-scoring network was Cell-To-Cell Signaling and Interaction, Hematological System Development and Function, and Inflammatory Response (score = 50). Red nodes indicate up-regulation; green nodes indicate down-regulation. Arrows and lines denote interactions between specific genes within the network. A, activation; E, expression regulation; I, inhibition; L, proteolysis; LO, localization; M, biochemical modification; MB, membership of a group or complex; P, phosphorylation; PD, protein-DNA interaction; PP, protein-protein interaction; PR, protein-RNA interaction; RB, regulation of binding; RE, reaction; T, transcription; TR, translocation.

To identify pathways regulated by HMGA1 early in tumorigenesis, we first analyzed the differentially expressed genes at 2 months identified by CAM using IPA (Table 3). From the list of differentially regulated genes, 5 pathways had a significant network score (≥ 3.0) and the pathways involved in cell-to-cell signaling and interaction, hematologic system development and function, and inflammatory response had the most significant network score (score = 50; Figure 1). In this network, *interferon beta *(*IFNβ*) is an important node, which functions as a cytokine in antiviral defense and was previously identified as a transcriptional target of HMGA1 [16,48-53]. *IFNβ *is involved in cell surface signaling, B cell proliferation, induction of apoptosis, and the positive regulation of innate immunity. *Interferon alpha *(*IFNα*) is another major node in this network, which also functions as a cytokine involved in antiviral defense. In addition, the Nuclear Factor-κB (NFκB) transcription factor complex was identified as a major node and members of this family are known to interact with HMGA1 and form a higher order transcriptional complex or enhanceosome that functions to regulate gene expression of specific transcriptional targets, such as *IFNβ *[16,48-53]. NFκB plays an important role in immune function and mediating inflammatory signals. NFκB also appears to have a central role in tumorigenesis, particularly in cancers linked to inflammation [54]. JNK, or the family of c-Jun N-terminal kinases, was also identified as a central node. JNKs were originally defined as kinases that phosphorylate c-Jun within its transcriptional activation domain and include the mitogen-activated protein kinases (MAPKs) [55]. These proteins belong to a family of serine/threonine kinases that modify the activities of nuclear and mitochondrial proteins through phosphorylation. MAPKs respond to stress stimuli and are involved in inflammation and cytokine production as well as regulating proliferation, survival, migration, metabolism, apoptosis, transcription and translation. The p38 MAPK complex was also identified as a node and includes MAPK11, MAPK12, MAPK13, and MAPK14. Like the JNK complex, these protein kinases also respond to extracellular stress stimuli, including cytokines, ultraviolet irradiation, heat shock, and osmotic changes [55,56], transducing extracellular signals into diverse cellular pathways involved in cellular homeostasis. The top disease-related function identified in the network was the inflammatory response while cellular development (which refers to cellular development and differentiation processes, including hematopoiesis, maturation and senescence) was the most the top biologic function and the most significant molecular and cellular function (Table 3). The most significant physiologic system development and function category from this network was the hematologic system, which includes normal development of all hematopoietic cells (Table 3).

**Table 3:** Top functions by pathway analysis (IPA)

	CAM	Partek	Overlaps	CAM	Partek	Overlaps
	2months	2months	2months	12months	12months	12months
**Disease & Disorder-related Function**	Inflammatory response	Inflammatory response	*Inflammatory response	Cancer	Cancer	Cancer
	3.57 × 10^-5 ^- 0.0320	1.61 × 10^-5 ^- 0.0474	1.93 × 10^-4 ^- 0.0474	2.71 × 10^-7 ^-6.96 × 10^-3^	2.84 × 10^-6 ^-3.44 × 10^-3^	1.77 × 10^-5 ^-.0133

**Molecular & Cellular Functions**	*Cellular development	*Cellular function & maintenance	Cell-to-cell signaling & interaction	*Cellular development	*Cell cycle	*Cell cycle
	3.58 × 10^-7 ^-0.0326	1.87 × 10^-6 ^- 0.0444	1.93 × 10^-4 ^- 0.0488	7.10 × 10^-13 ^-7.58 × 10^-3^	1.23 × 10^-11 ^- 3.09 × 10^-3^	2.32 × 10^-8 ^-.0133

**Physiological System Development & Function**	Hematological system development & function	Hematological system development & function	Hematological system development & function	Hematological system development & function	Cell-mediated immune response	Hematological system development & function
	3.06 × 10^-5 ^-0.0326	2.62 × 10^-6 ^-0.0474	1.93 × 10^-4 ^-0.0485	1.89 × 10^-12 ^-7.26 × 10^-3^	1.27 × 10^-7 ^-3.09 × 10^-3^	3.02 × 10^-6 ^-.0133

The top functions identified by Ingenuity Pathway Analysis from each category are shown and include: 1.) Diseases and Disorder-related Function, 2.) Molecular and Cellular Functions, and, 3.) Physiological System Development and Function. * denotes the top biologic function, defined as the function from all 3 categories with the most significant IPA score.

#### Parametric analysis

**Figure 2:**
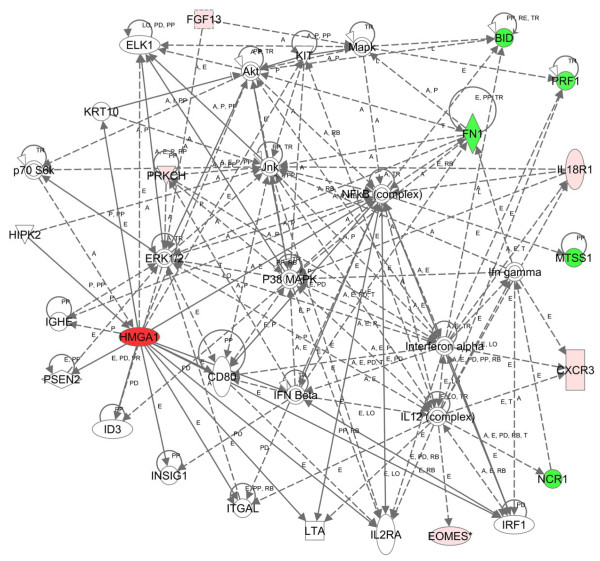
**Network of differentially expressed genes in *HMGA1 *transgenics at 2 months (parametric approach)**. Using 36 differentially expressed genes as the focus gene set identified by a parametric approach and IPA, the highest-scoring network was Immunological Disease, Cell-Mediated Immune Response, and Cellular Development (score = 23). Colors, arrows, lines and abbreviations are described under Figure 1.

We also analyzed the differentially expressed genes at 2 months identified by Partek with IPA. From the list of differentially regulated genes, there were 6 significant networks, and the most significant network (score = 23; Figure 2) was involved in immunological disease, cell-mediated immune response, and cellular development. In this network, important nodes include 1.) ERK 1/2 complex (including MAPK1 and MAPK2) which function in responding to cellular stress or DNA damage and induce apoptosis, chemotaxis, migration, proliferation, and inhibit differentiation [56], and, 2.) the interleukin 12 complex (including IL12A and IL12B), which function as pro-inflammatory cytokines involved in T-cell mediated cytotoxicity, natural killer cell-mediated cytotoxicity, the immune response, cell cycle arrest, migration, and T cell and B lymphocyte proliferation [57]. Similar to the IPA analysis of the CAM data at 2 months, JNK, P38 MAPK, INFβ, and NFκB were also identified as important nodes. Cellular function and maintenance (which describes functions associated with the maintenance of cellular homeostasis, such as metabolism, respiration, DNA repair, endoplasmic reticulum stress response, exocytosis, autophagy, and phagocytosis), was the top biologic function and the most significant molecular and cellular function. As with the nonparametric analysis, the inflammatory response was the most significant disease-related function and the hematological system was the most significant physiologic system function (Table 3). Differentially expressed genes with a p value of ≤ 0.01 were also assessed by IPA and identified pathways involved in gene expression, cell death, and hematologic development and function (see Additional file 1).

### Analysis of concordant genes identified by the parametric and nonparametric approaches

**Figure 3:**
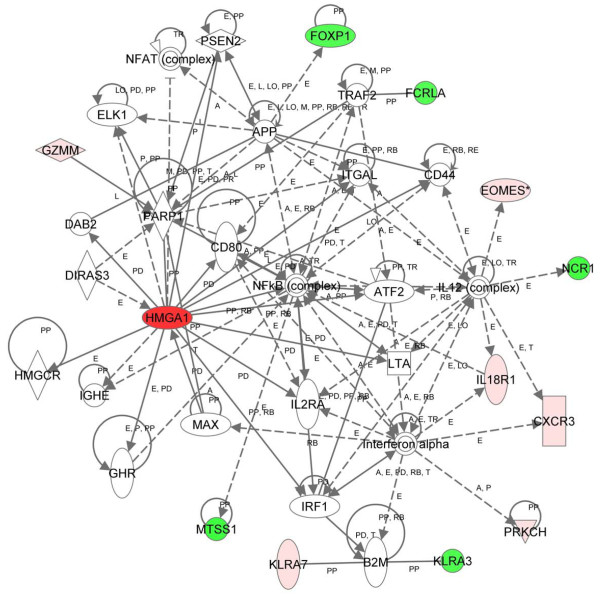
**Network of differentially expressed genes in transgenics at 2 months (parametric and nonparametric approaches)**. Using 21 differentially expressed genes as the focus gene set from the overlap of genes identified from the parametric and the nonparametric approaches and IPA, the highest-scoring network was Immunological Disease, Cellular Development, and Cellular Function and Maintenance (score = 30). Colors, arrows, lines and abbreviations are described under Figure 1.

Finally, we analyzed the overlap of the differentially expressed genes identified using the nonparametric CAM and the parametric Partek approaches at 2 months with IPA. There were 4 significant networks, and the most significant network (score = 30; Figure 3) was involved with immunological disease, cellular development, and cellular function and maintenance. In this network, the NFκB and interleukin 12 complexes were important nodes. The inflammatory response was the top biologic function and the most significant disease-related function. Cell-to-cell signaling interaction was the most significant molecular and cellular function (Table 3). As before, the hematologic system was the most significant physiologic system function (Table 3). Of note, the *metastasis suppressor protein 1 *(*MTSS1*) gene was repressed and appears as a node in networks from all three analyses early in tumorigenesis (Figures 1, 2, 3). In addition, the *EOMES *(*eomesodermin*) stem cell gene was up-regulated and identified as a node from all three analyses. The *EOMES *gene plays a crucial role in embryogenesis during both trophoblastic development and gastrulation. It is also involved in brain development and the differentiation of CD8+ T cells.

### The transcriptional networks dysregulated by HMGA1 late in tumorigenesis

#### Nonparametric analysis

**Figure 4:**
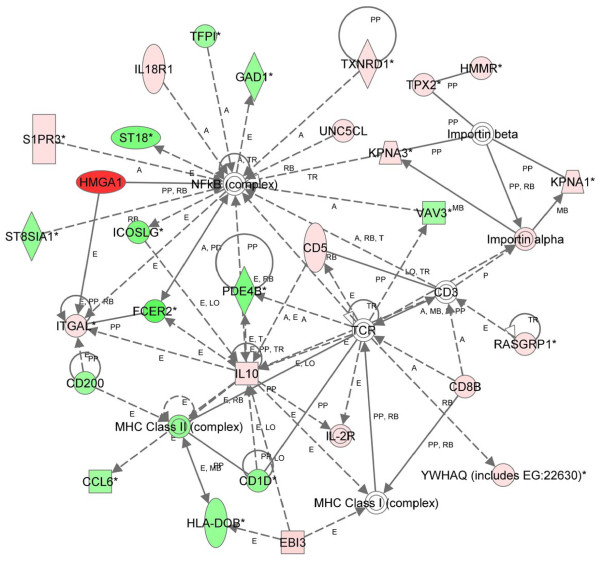
**Network of differentially expressed genes in *HMGA1 *transgenics at 12 months (nonparametric approach)**. Using 425 differentially expressed genes as the focus gene set identified from a nonparametric approach and IPA, the highest-scoring network was Inflammatory Response, Immunological Disease, and Neurological Disease (score = 44). Colors, arrows, lines and abbreviations are described under Figure 1.

From the list of differentially expressed genes identified by CAM at 12 months, there were 21 significant networks, and the pathway with the most significant network (score = 44) was involved in inflammatory response, immunological disease, and neurological disease (Figure 4). In this network, important nodes included: 1.) interleukin 10, an anti-inflammatory cytokine induced by inflammation, cellular stress, and the immune response which down-regulates Th1 cytokines and enhances B cell survival, proliferation, and antibody production [58], 2.) T-cell receptor complex, which is involved in T-cell activation [58,59], and, 3.) the NFκB complex, which was also a prominent node in the pathway analyses at 2 months. In contrast to the pathways identified early in tumorigenesis, the top disease-related function was cancer. Cellular development was the top biologic function and the most significant molecular and cellular function. As with the analysis early in tumorigenesis, the hematological system was the most significant physiologic system function (Table 3).

#### Parametric analysis

**Figure 5:**
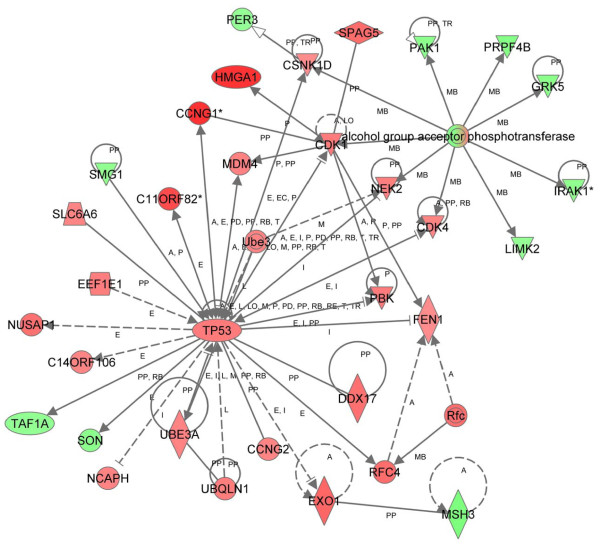
**Network of differentially expressed genes in *HMGA1 *transgenics at 12 months (parametric approach)**. Using 715 differentially expressed genes as the focus gene set, identified by a parametric approach and IPA, the highest-scoring network was DNA Replication, Recombination and Repair, Cancer, and Cell Cycle (score = 48). Colors, arrows, lines and abbreviations are described under Figure 1.

There were 29 significant networks identified from the list of differentially expressed genes at 12 months identified by Partek and the most significant network (score = 48) was involved in DNA replication, recombination, and repair, cancer, and cell cycle (Figure 5). In this network important nodes included: 1.) The TP53 (tumor protein 53) tumor suppressor, which is mutated in many cancers and involved in apoptosis, genomic instability, inhibition of angiogenesis, and cell cycle arrest [60], 2.) CDK1 (cyclin dependent kinase 1 or cell division control protein 2 homolog), which is a highly conserved serine/threonine kinase that is a key regulator of cell cycle progression [61], and, 3.) the alcohol group acceptor phosphotransferases, which include multiple proteins such as MAPKs, protein kinases, and cyclin dependent kinases [55,56]. As above, cancer was the most significant disease-related function. Cell cycle was the top biologic function and the most significant molecular and cellular function. Cell-mediated immune response was the most significant physiological system function (Table 3). Differentially expressed genes with a p value of ≤ 0.01 were also assessed by IPA and identified pathways involved in cancer, gene expression, and reproductive system disease (see Additional file 1).

### Analysis of concordant genes identified by parametric and nonparametric approaches

**Figure 6:**
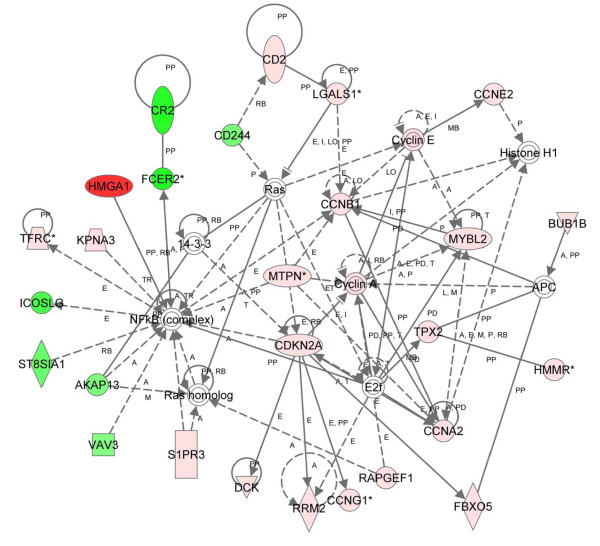
**Network of differentially expressed genes in transgenics at 12 months (parametric and nonparametric approaches)**. Using 117 differentially expressed genes as the focus gene set identified by both a parametric and nonparametric approach and IPA, the highest-scoring network was Cell Cycle, Cellular Development, and Cellular Growth and Proliferation (score = 58). Colors, arrows, lines and abbreviations are described under Figure 1.

We identified 6 significant networks from the overlap of the differentially regulated genes at 12 months with the most significant network score (score = 58) involved in cell cycle, cellular development, and cellular growth and proliferation (Figure 6). In this network, important nodes included: 1.) cyclin B1, which regulates the G2/M transition in cell cycle progression, mitosis, apoptosis, checkpoint controls, growth and maturation, and is up-regulated in diverse cancers [62], 2.) cyclin A (including cyclin A1 and A2), which regulates cell cycle progression, apoptosis, and checkpoint controls and is also elevated in diverse tumors [63], 3.) the E2F complex, which includes important regulators of proliferation and cell cycle progression [64], and, 4.) *CDKN2A *tumor suppressor, which is involved in apoptosis, cell cycle arrest, and senescence [65]. The *CDKN2A *locus is deleted or silenced in many forms of cancer, including T- and preB cell leukemia [65]. As before, NFκB was a central node. Cell cycle progression was the top biologic function and the most significant molecular and cellular function. Cancer was the most significant disease-related function and the hematological system was the most significant physiologic system function (Table 3).

### Validation of selected genes dysregulated by *HMGA1*

To validate our microarray gene expression profile analysis, we performed qRT-PCR for candidate genes identified as differentially expressed by microarray analysis (Table 4). At the early time point in tumorigenesis (2 months), we assessed 6 genes and confirmed differential expression of all 6 (Table 1), with 5 up-regulated (*CXCR3*, *EOMES*, *IL18R1*, *GZMM*, *IL2rb*) and 1 down-regulated (*FOXP1*). Moreover, the fold-changes of these genes by microarray analysis and qRT-PCR were similar (Table 4). At the 12 month time point, we assessed expression of 7 candidate genes and confirmed differential expression in all cases. Six genes were up-regulated (*BUB1*, *EOMES*, *IL18R1*, *GZMM*, *IL2rb*, *CD8b1*) and one (*FOXP1*) was down-regulated. Many of these genes function in inflammation (*CXCR3*, *EOMES*, *IL18R1*, *GZMM*, *IL2rb*) [66], metastases (*CXCR3*, *BUB1*) [66-68], poor outcome cancers (*EOMES*, *BUB1*) [67,68], or stem cells (*EOMES*, *BUB1*) [67-71]. In addition, a subset of these genes have been identified as markers for poor outcomes in lymphoid and other cancers *(CXCR3*, *EOMES*, *BUB1*) [66-68]. Thus, our validation studies confirmed the differential expression of all of the genes we assessed (Table 4).

**Table 4:** Comparison of microarray and qRT-PCR results for selected genes

Early/2 months	Microarray	qRT-PCR	
**Gene Symbol**	**Fold-change**	**Fold-change**	**p-value**

*Cxcr3*	1.534	1.604	7.13E-05

*Eomes*	1.460	1.610	1.29E-02

*FoxP1*	-1.326	-1.512	3.30E-04

*Gzmm*	4.297	11.452	5.41E-06

*IL18r1*	1.399	2.096	2.15E-03

*IL2rb*	1.566	1.415	6.03E-03
**Late/12 months**	**Microarray**	**qRT-PCR**	

**Gene Symbol**	**Fold-change**	**Fold-change**	**p-value**

*Bub1b*	1.618	1.423	3.82E-07

*CD8b1*	2.541	1.889	2.76E-02

*Eomes*	2.327	2.073	1.28E-02

*FoxP1*	-2.593	-2.045	9.80E-05

*Gzmm*	10.424	24.514	8.60E-03

*IL18r1*	2.085	2.042	2.88E-02

*IL2rb*	1.821	1.516	3.61E-02

A subset of genes identified as differentially expressed by microarray analysis were validated by qRT-PCR at both the early and late time points. The fold-change found by microarray and qRT-PCR were similar for all genes assessed by qRT-PCR.

### HMGA1 transcriptional targets found in cells from human lymphoid tumors

To determine if any of the genes dysregulated by HMGA1 in the transgenic model are relevant to human lymphoid malignancy, we knocked-down HMGA1 in Jurkat cells (a human T-cell acute lymphoblastic leukemia or T-ALL cell line) and assessed the expression of the 8 genes validated in the transgenic model by qRT-PCR (see Additional file 2). We found that 3 of the 8 genes were also significantly down-regulated in the Jurkat T-ALL cells, including *EOMES*, *IL2RB, and CD8B1; *see Additional file 2). These data indicate that a subset of the genes dysregulated by HMGA1 in our transgenic model are also relevant in human T-ALL cells.

### Geneset enrichment analysis

To determine if the gene expression profiles identified in our study overlap with previously reported gene sets, the Molecular Signatures Database (http://www.broad.mit.edu/gsea) was interrogated [46,47]. We found that the gene set derived from up-regulated genes in the established tumors by both the parametric and nonparametric approaches had the most significant overlaps (p ≤ 10^-8^) with previously published gene sets (Table 5). The three gene sets with p ≤ 10^-8 ^included progenitor T cells [72], embryonic stem cells [73], and neural stem cells [73]. The gene set with the highest significant overlap (LEE T CELLS 3 UP; p < 2.26E-09) was derived from a gene expression profile study of human intrathymic T cells at different stages in maturation [73]. The overlapping gene set from this study was from transcripts enriched in intrathymic T progenitors (ITTP) and double positive (DP or CD3+/CD4+) T cells compared to more mature T cells. These results indicate that the *HMGA1 *tumors share gene expression profiles with the DP, less differentiated, thymic progenitors. The second gene set with highly significant overlap (EMBRYONIC STEM CELL UP; p < 5.53E-09) was derived from a study of gene expression profiles in mouse embryonic stem cells compared to differentiated brain and bone marrow cells [73]. Similarly, the third gene set was derived from the same study (NEURAL STEM CELL UP; p < 7.22E-09) and included genes enriched in neural stem cells compared to differentiated brain and bone marrow cells [73]. Not surprisingly, this prior study reported overlap between the embryonic and neural stem cell gene sets. These results suggest that HMGA1 drives transcriptional networks that promote the maintenance of a poorly differentiated, stem-like phenotype in our transgenic model.

**Table 5:** Gene sets that overlap with the HMGA1 gene sets

Up-regulated Genes in Established Tumors	
**Gene set name**	**p value**

LEE TCELLS3 UP	2.26E-09

STEMCELL EMBRYONIC UP	5.53E-09

STEMCELL NEURAL UP	7.22E-09

The Molecular Signature Database was interrogated for overlaps with the gene sets identified in our analyses. The most significant overlaps (p ≤ 10^-8^) are shown above with the relevant p value. (See Additional file 4 for all overlaps identified.)

## Discussion

Here, we report for the first time cellular pathways induced by HMGA1 at different stages of tumorigenesis. Despite its widespread overexpression in virtually all poorly differentiated tumors and aggressive hematologic malignancies studied to date [1-23], there were no prior studies to identify transcriptional targets dysregulated by HMGA1 during tumor progression. Moreover, fewer than 50 bona fide HMGA1 transcriptional targets have been reported to date [16-18]. The *IFNβ *gene is the most extensively studied downstream gene target of HMGA1. HMGA1 binds to the 5' UTR of this gene and promotes cooperative binding of NFκB (p50/p65) and additional transcription factors [48-53]. Together with these factors, HMGA1 bends DNA and forms a transcriptional enhancer complex or "enhanceosome" that is essential for the expression of this gene. Emerging evidence indicates that HMGA1 is a key transcription factor in embryonic stem cells and diverse tumors with poor outcomes [1]. These findings suggest that HMGA1 drives a refractory, stem-like state in cancer by inducing specific genes and their cellular pathways. Hence, studies to identify the HMGA1 transcriptome during tumorigenesis should have important implications, not only for cancer, but also for development and the stem cell phenotype.

Early in tumor development, we found that genes involved in the inflammatory response are dysregulated by HMGA1. NFκB was identified as a key node involved in HMGA1 signaling, consistent with prior studies demonstrating that HMGA1 forms an enhanceosome with NFκB [48-53]. Our results also indicate that NFκB is intimately involved with malignant transformation in this model, both early in tumor initiation, and later, in established tumors. Notably, most of the previously reported HMGA1 transcriptional targets include NFκB regulatory elements in their promoter regions and many participate in mediating inflammatory pathways [[16], Resar and Sumter unpublished data]. Given the recent evidence that inflammation is a precursor lesion in some cancers, this link between HMGA1, NFκB, and inflammation are consistent with a model whereby these proteins cooperate to induce inflammatory signals and drive transformation. This result also opens up potential avenues for therapeutic and even preventive interventions to block tumor initiation and/or progression. In preliminary studies, we found that crossing the *HMGA1 *transgenics with mice null for the p50 subunit of NFκB results in an increase in lymphoid tumor burdens (Belton & Resar, unpublished data). The NFκB transcriptional complex is comprised primarily of p65:p50 heterodimers in which p65 functions as an activator, and p50, which lacks a transcriptional activation domain, is thought to function as an inhibitor. The p50/p65 heterodimers are thought to activate transcription of canonical NFκB genes, whereas p50 homodimers repress transcription. Indeed, mice deficient in p50 have been shown to have enhanced NFκB activity and inflammatory responses [74]. Although further confirmatory studies are needed, our preliminary results suggest that excess NFκB activation (with loss of inhibition by p50) could lead to enhanced tumorigenesis induced by HMGA1.

The up-regulation of inflammatory pathways during HMGA1-induced tumorigenesis is also consistent with our previous studies demonstrating that anti-inflammatory agents (sulindac and celecoxib) interfere with sarcomas in our *HMGA1 *transgenics [12]. The anti-inflammatory drugs did not significantly affect the lymphoid tumors, however, suggesting that *COX-2 *may not be consistently up-regulated in these tumors. Indeed, we assessed *COX-2 *expression in the lymphoid tumors and found up-regulation in only 50% of lymphoid cells early in tumorigenesis; it was not up-regulated in the established tumors (see Additional file 3). Moreover, the tumor size was decreased in ~50% of the treated tumors, suggesting that the inhibitors function only in tumors up-regulating *COX-2*. We also identified the *IFNβ *gene as a node involved in HMGA1 function early in tumorigenesis, which further validates both our model system and experimental approach because this is an established HMGA1 target gene. Moreover, we uncovered additional cytokine nodes that further support the role of HMGA1 in driving inflammatory pathways during lymphoid tumorigenesis, including *IFNα *and *IL-12*. In addition, *JNK*, *ERK1/2 *and *MAP kinases *were key nodes early in tumorigenesis, additional pathways that could be pharmacologically manipulated in therapy. Interestingly, all networks from this early time point show down-regulation of the *MTSSI *gene, which encodes the metastasis suppressor protein 1. Repressing a suppressor of metastases represents a previously undescribed pathway through which HMGA1 promotes tumor progression. The *EOMES *gene was also up-regulated in all networks and this gene is crucial in embryonic development. Previous studies also show that *EOMES *are overexpressed in colorectal cancer [75].

Later in tumorigenesis, we identified genes involved in cell cycle progression as a central pathway. Although HMGA is thought to induce cellular proliferation in some settings [16], its role in cell cycle progression has not been extensively studied and warrants further investigation, particularly because cell cycle inhibitors are available and newer agents are under development. Our results suggest that HMGA1 up-regulates expression of the E2F proliferation genes, and at least three key cyclins or cyclin complexes, including the cyclin A complex, cyclin B1, and cyclin E, the latter of which is a downstream transcriptional target of E2F1. These findings are consistent with previous studies in adipocytes expressing a carboxyl-terminal, truncated HMGA1 that showed increased E2F1 protein levels and E2F1 DNA binding activity [76]. Subsequent studies demonstrated that HMGA1 interacts with the RB protein and interferes with RB-mediated repression of E2F1 transcription and cell cycle progression in neuroblastoma cells [77]. Thus, HMGA1 could promote tumor progression by inducing a molecular program for cell cycle progression and proliferation with up-regulation of E2F1 and cyclins. CDK1 was also identified as a node in the parametric analysis of differentially expressed genes in the established tumors, which could further augment cyclin function through activation by phosphorylation. The TCR complex was identified as a node in the nonparametric analysis and functions in T cell activation and function. Interestingly, *HMGA1 *null mice have a paucity of T cells, further substantiating an important role for HMGA1 in T cell development and function [78]. In addition, two prominent tumor suppressor loci were identified as major nodes in the established tumors, namely *CDKN2A *and *TP53*. Notably, the *CDKN2A *locus is deleted in up to 90% of cases of T-cell leukemia [65], while this locus is silenced through methylation in other forms of leukemia. The *CDKN2A *locus encodes the Ink4a and Arf tumor suppressor proteins which function upstream of Rb and p53, respectively, and serve to maintain the tumor suppressor function of Rb and p53. It is not clear if *CDKN2A *is mutated in our mouse tumors, although we have preliminary evidence that expression of at least the *INK4A *is decreased in a subset of the lymphoid tumors (Resar, unpublished data). *TP53*, the other tumor suppressor node identified in our analysis, is among the most frequently mutated tumor suppressors in cancer with mutations occurring in most human tumors. TP53 functions by helping cells respond to DNA damage and other cellular stresses, acting as a "gate keeper" of the integrity of the genome [60]. It is possible that TP53 is inactivated through INK4A/ARF mutation or inactivation in our tumor model, similar to Myc-induced lymphoid tumors in mice [79,80]. Future studies are needed to assess the functional status of these tumor suppressors in the HMGA1 transcriptional networks and are likely to uncover additional pathways that could be targeted in hematologic and other malignancies driven by HMGA1.

We also found that gene sets regulated by HMGA1 overlap with stem cell gene sets, including embryonic and neural stem cells compared to differentiated cells. The gene set enrichment analysis also showed overlap with immature T cells. These results indicate that HMGA1 drives transcriptional pathways shared by poorly-differentiated, stem-like cells. Because prior studies demonstrate that cancers with stem-like molecular signatures have poor outcomes [1], it is likely that HMGA1 drives a refractory, advanced tumor by inducing these pathways in tumor cells. HMGA1 is also highly expressed during embryogenesis and it may regulate similar cellular pathways during development and promote tumorigenesis when these pathways are re-activated in the postnatal period.

## Conclusions

Here, we found that *HMGA1 *induces genes involved in inflammatory pathways early in lymphoid tumorigenesis and genes involved in embryonic stem cells, cell cycle progression, and proliferation in established tumors. HMGA1 also dyregulates genes and pathways that function in stem cells, cellular development and hematopoiesis at both early and late stages of tumorigenesis. These results provide insight into HMGA1 function at different stages in tumor development and point to cellular pathways that could serve as therapeutic targets in lymphoid and other human cancers with aberrant *HMGA1 *expression.

## Abbreviations

*HMGA1a: high mobility group A1 *gene; qRT-PCR: real time polymerase chain reaction

## Authors' contributions

LMSR conceived and designed the experiments and wrote the final version of the paper, AS generated the networks and performed the gene set enrichment analysis, wrote the results section and prepared figures and tables, AB prepared samples, tables and figures, conceived, designed and performed the knock-down experiments and gene expression studies, JK and CCT conceived, designed, and performed the statistical analysis of the gene expression data, FPD prepared the mouse samples and validated differential expression of *HMGA1 *in these samples, WP performed gene expression analysis from the mouse samples, HLS performed the nonparametric statistical analysis, SNS performed gene expression studies in human leukemia cells, THH performed gene expression analysis in the mouse and human samples, DLH designed experiments related to the mouse model and sample preparation. All authors read and approved the final manuscript.

## Supplementary Material

Additional file 1 **Table and networks of differentially expressed genes with p ≤ 0.01**. **A.) **Table of all differentially expressed genes in the HMGA1 mice compared to controls at 2 months and 12 months with a p ≤ 0.01. **B.) **Network of differentially expressed genes identified by a parametric approach in the *HMGA1 *transgenic mice at 2 months with a p ≤ 0.01. **C.) **Network of differentially expressed genes identified by a parametric approach in the *HMGA1 *transgenic mice at 12 months with a p ≤ 0.01. Click here for file

Additional file 2 **Data from HMGA1 knock-down in human leukemia cells (Jurkat T-cell ALL cells)**. Data from knock-down of HMGA1 in Jurkat cells are shown, including mRNA and protein expression for HMGA1 and mRNA expression for *CD8B1*, *EOMES*, and *IL2RB.* Click here for file

Additional file 3 **COX-2 data from HMGA1 and control mice**. *COX-2 *mRNA expression in the spleens from *HMGA1 *or control mice at 2 months is shown. Click here for file

Additional file 4 **Gene set enrichment analysis**. This file includes all overlaps with our gene sets identified by gene set enrichment analysis. Click here for file
