# Supplementary material for: HMGA1 drives stem cell, inflammatory pathway, and cell cycle progression genes during lymphoid tumorigenesis
Source: BMC Genomics. 2011 Nov 4;12:549. doi: 10.1186/1471-2164-12-549 (PMC3245506; doi:10.1186/1471-2164-12-549)

A.) Table of differentiatially expressed genes in HMGA1 transgenics comparted to controls with p $\leq$ 0.01.

2 month up (7)
eomesodermin homolog (Xenopus laevis)
fibroblast growth factor 13
Way 5' of Cntn3, contactin 3 precursor
regulator of G-protein signaling 17
G protein-coupled receptor 15
natural killer cell group 7 sequence
RIKEN cDNA 9530034A14 gene

2 month down (7)
killer cell lectin-like receptor subfamily A, member 9
RNA binding motif protein 25
CD209b antigen
death associated protein-like 1
Fc receptor-like A
cDNA sequence BC032203
TruB pseudouridine (psi) synthase homolog 2 (E. coli)

12 month up (63)
aryl-hydrocarbon receptor
cyclin A2
cyclin B2
cyclin G1
glucosyltransfera
zinc finger protein 106
aurora kinase A
ceruloplasmin
centromere protein E
GrpE-like 1, mitochondrial
NudC domain containing 1
transmembrane protein 33
M-phase phosphoprotein 8
integrator complex subunit 7
diacylglycerol kinase, delta
kinesin family member 18A
CUG triplet repeat, RNA binding protein 1
nuclear receptor subfamily 3, group C, member 1
general transcription factor II I
hemoglobin Z, beta-like embryonic chain
insulin-like growth factor binding protein 2
lectin, galactose binding, soluble 1
AF4/FMR2 family, member 1
myeloblastosis oncogene-like 2
cyclin-dependent kinase inhibitor 2A
chloride channel, nucleotide-sensitive, 1A
eukaryotic translation initiation factor 2-alpha kinase 2
twinfilin, actin-binding protein, homolog 1 (Drosophila)
transcription elongation factor A (SII) 1
ATPase, H+ transporting, lysosomal V0 subunit A2
thymidylate synthase
ubiquitin-conjugating enzyme E2A, RAD6 homolog (S. cerevisiae)
ring finger protein 13
eukaryotic translation initiation factor 4E member 2
torsin family 1, member B
regulator of G-protein signaling 3
KRR1, small subunit (SSU) processome component, homolog (yeast)
myotubularin related protein 1
NTF2-related export protein 1
golgi SNAP receptor complex member 2
ribulose-5-phosphate-3-epimerase
lectin, mannose-binding 2
DEAD (Asp-Glu-Ala-Asp) box polypeptide 17
polymerase (DNA-directed), delta 3, accessory subunit
required for meiotic nuclear division 5 homolog A (S. cerevisiae)
RIKEN cDNA 1110018G07 gene
integrator complex subunit 2
WD repeat domain 68
establishment of cohesion 1 homolog 2 (S. cerevisiae)
spindle assembly 6 homolog (C. elegans)
RIKEN cDNA 4930547N16 gene
mitochondrial ribosomal protein L9
transducin (beta)-like 1X-linked receptor 1
proteasome (prosome, macropain) activator subunit 4
casein kinase 1, delta
extra spindle poles-like 1 (S. cerevisiae)
Rap guanine nucleotide exchange factor (GEF) 1
jumonji domain containing 6
SFT2 domain containing 2
acyl-Coenzyme A binding domain containing 3
3-phosphoglycerate dehydrogenase
mannoside acetylglucosaminyltransferase 4, isoenzyme A
asparagine-linked glycosylation 10 homolog B (yeast, alpha-1,2-
RIKEN cDNA 0610010B08 gene

12 month down (89)
poly (ADP-ribose) polymerase family, member 1
Rho GTPase activating protein 6
BCL2-like 2
oviductal glycoprotein 1
InaD-like (Drosophila)
clusterin
cannabinoid receptor 2 (macrophage)
dynein cytoplasmic 1 heavy chain 1
estrogen receptor 1 (alpha)
Ecotropic viral integration site 2a, mRNA (cDNA clone MGC:47928 IMAGE:1396641)
Fc receptor, IgE, low affinity II, alpha polypeptide
guanine nucleotide binding protein, alpha q polypeptide
G protein-coupled receptor kinase 5, mRNA (cDNA clone MGC:30358 IMAGE:5116510)
histocompatibility 2, O region beta locus
interleukin 3 receptor, alpha chain
kallikrein 1
LIM motif-containing protein kinase 2
myocyte enhancer factor 2C
nuclear factor of kappa light polypeptide gene enhancer in B-cells 2, p49/p100
CD244 natural killer cell receptor 2B4
Intron of Npr1, natriuretic peptide receptor 1 precursor
Intron or possible novel exon of Pax5, paired box gene 5
RAS, guanyl releasing protein 2
Intron of Ncoa3, nuclear receptor coactivator 3
nuclear antigen Sp100
titin
homer homolog 3 (Drosophila)
cytokine receptor-like factor 3
Mediterranean fever
cell adhesion molecule 1
zinc finger and BTB domain containing 20
vav 3 oncogene
nischarin
serine (or cysteine) peptidase inhibitor, clade B, member 1a
transcription factor 25 (basic helix-loop-helix)
Possible extended 3'UTR of Cdc37l1, cell division cycle 37 homolog (S. cerevisiae)-like 1
RIKEN cDNA 4932415G12 gene
RIKEN cDNA 1110029L17 gene
putative homeodomain transcription factor 2
hydroxyacylglutathione hydrolase-like
RIKEN cDNA 1500032P08 gene
ubiquitin protein ligase E3 component n-recognin 4
RAB43, member RAS oncogene family
solute carrier family 25, member 33
arrestin domain containing 2
sperm associated antigen 9
RIKEN cDNA 4933407H18 gene
Intron of Arhgap26, Rho GTPase activating protein 26
zinc finger and BTB domain containing 46
RIKEN cDNA 2510009E07 gene
metastasis associated lung adenocarcinoma transcript 1 (non-coding RNA)
MTERF domain containing 3
Intron of 1700081L11Rik, RIKEN clone
RIKEN cDNA 9530053H05 gene
helicase with zinc finger domain
opposite strand transcription unit to Stag3
solute carrier family 12 (potassium/chloride transporters), member 9
bromodomain and WD repeat domain containing 1
oxysterol binding protein-like 9
WD repeat domain 91
guanosine monophosphate reductase 2
O-linked N-acetylglucosamine (GlcNAc) transferase (UDP-N-acetylglucosamine:polyp
Forkhead box P1 (Foxp1), mRNA
ankyrin repeat domain 39
RNA binding motif protein 39
B and T lymphocyte associated
zinc finger protein 658
zinc finger protein 273
Intron of Arhgef11, Rho guanine nucleotide exchange factor (GEF) 11
Intron of Nav1, neuron navigator 1
Intron of Ccm2, cerebral cavernous malformation 2 homolog
Intron of Aftph, aftiphilin
zinc finger, MYM-type 5
kelch-like 22 (Drosophila)
RAP1, GTP-GDP dissociation stimulator 1
TAR DNA binding protein
RIKEN cDNA B930041F14 gene
myeloid/lymphoid or mixed-lineage leukemia 3
ATPase, class V, type 10D
SMG1 homolog, phosphatidylinositol 3-kinase-related kinase (C. elegans)
ubiquitin specific peptidase 3
Rho GTPase activating protein 22
B-cell scaffold protein with ankyrin repeats 1
ER lipid raft associated 2
RIKEN cDNA A230050P20 gene
hypothetical protein 9530028C05
hypothetical protein 3830612M24
aldo-keto reductase family 1, member C12
deleted in lymphocytic leukemia,

Gene names of all the genes selected by a parametric (Partek) approach with a fold-change (up or down) $\geq$ 1.3 and a p $\leq$ 0.01.

B.) Network of differentially expressed genes selected by a parametric approach in *HMGA1* transgenic mice at 2 months. IPA was performed on microarray data comparing transgenic to control lymphoid cells from mice. Using 14 differentially expressed genes as the focus gene set, identified by a parametric approach including p ≤ 0.01, the highest-scoring network was Gene Expression, Cell Death, and Hematological System Development and Function (score = 24). Colors, arrows, lines and abbreviations are described under Figure 1.

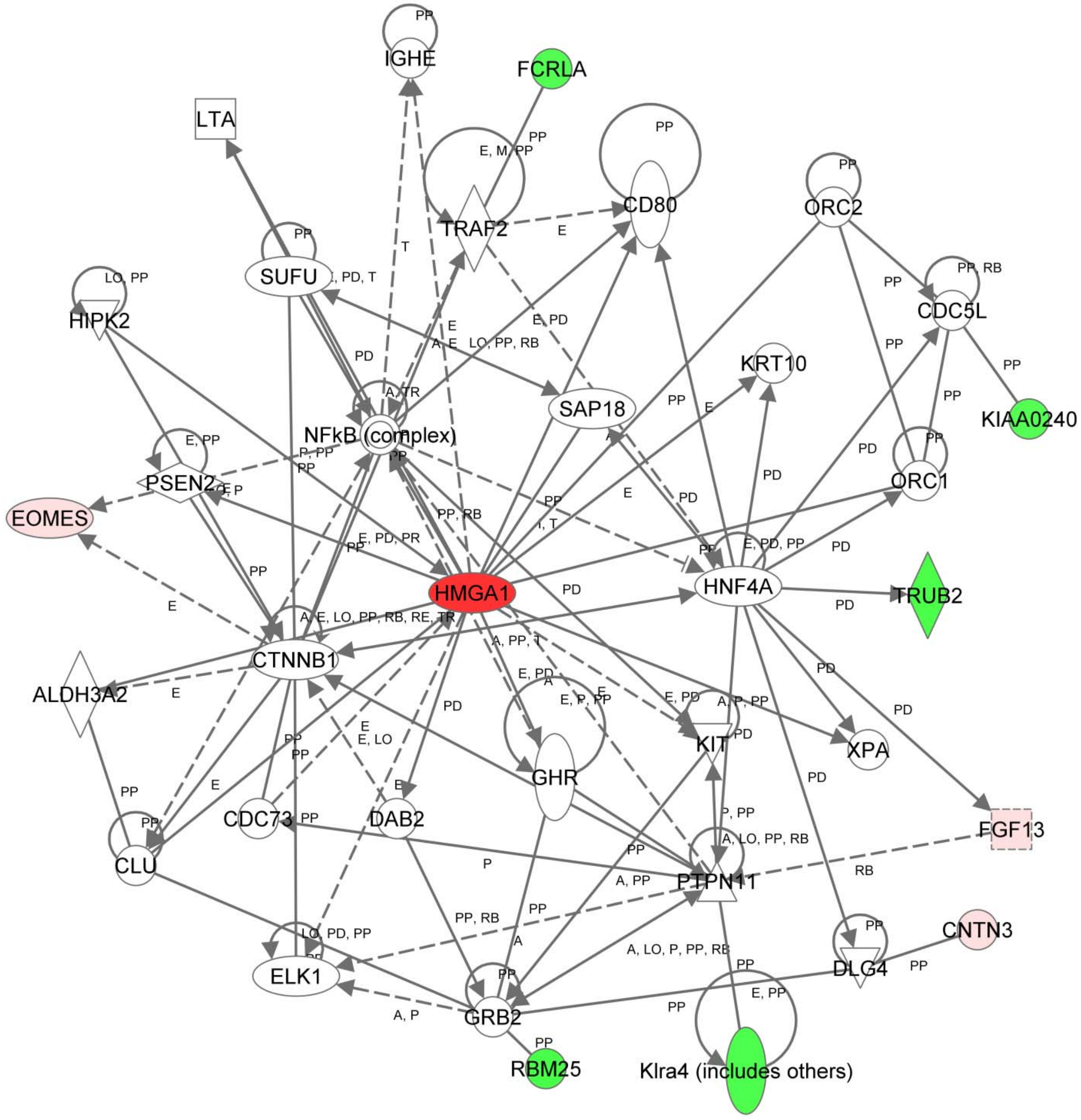

C.) Network of differentially expressed genes selected by a parametric approach in *HMGA1* transgenic mice at 12 months. IPA was performed on microarray data comparing transgenic to control lymphoid cells from mice. Using 152 differentially expressed genes as the focus gene set, identified by a parametric approach including $p \leq 0.01$, the highest-scoring network was Gene Expression, Cancer, and Reproductive System Disease (score = 48). Colors, arrows, lines and abbreviations are described under Figure 1.

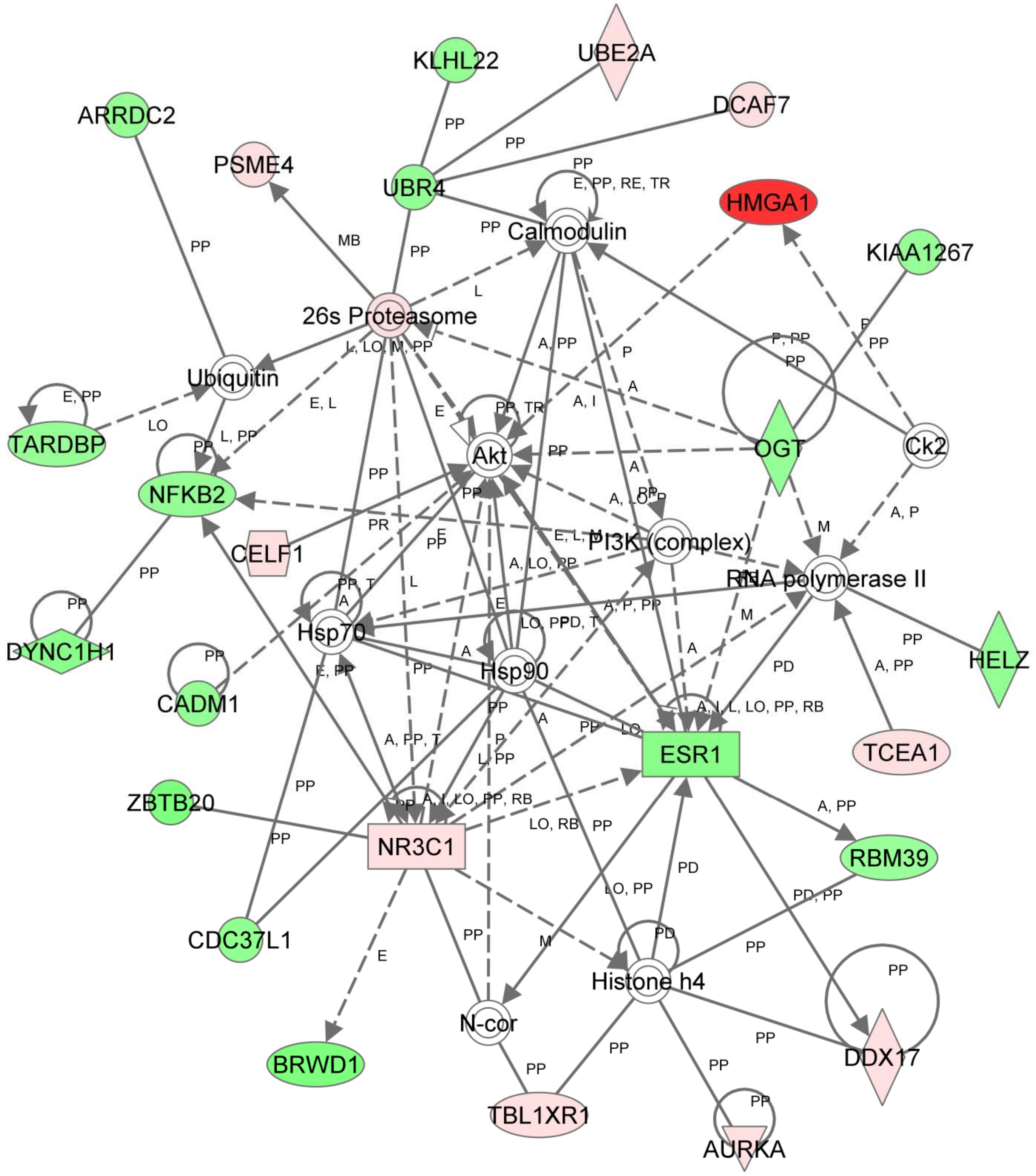

Supplement: Additional file 1 — Table and networks of differentially expressed genes with p ≤ 0.01. A.) Table of all differentially expressed genes in the HMGA1 mice compared to controls at 2 months and 12 months with a p ≤ 0.01. B.) Network of differentially expressed genes identified by a parametric approach in the HMGA1 transgenic mice at 2 months with a p ≤ 0.01. C.) Network of differentially expressed genes identified by a parametric approach in the HMGA1 transgenic mice at 12 months with a p ≤ 0.01. [file 1471-2164-12-549-S1.PDF]
