# Supplementary material for: HMGA1 drives stem cell, inflammatory pathway, and cell cycle progression genes during lymphoid tumorigenesis
Source: BMC Genomics. 2011 Nov 4;12:549. doi: 10.1186/1471-2164-12-549 (PMC3245506; doi:10.1186/1471-2164-12-549)
Supplement: Additional file 2 — Data from HMGA1 knock-down in human leukemia cells (Jurkat T-cell ALL cells). Data from knock-down of HMGA1 in Jurkat cells are shown, including mRNA and protein expression for HMGA1 and mRNA expression for CD8B1, EOMES, and IL2RB. [file 1471-2164-12-549-S2.PDF]

Knock-down of HMGA1 in human leukemia cells results in down-regulation in *CD8B1*, *EOMES*, and *IL2RB*.

A.)

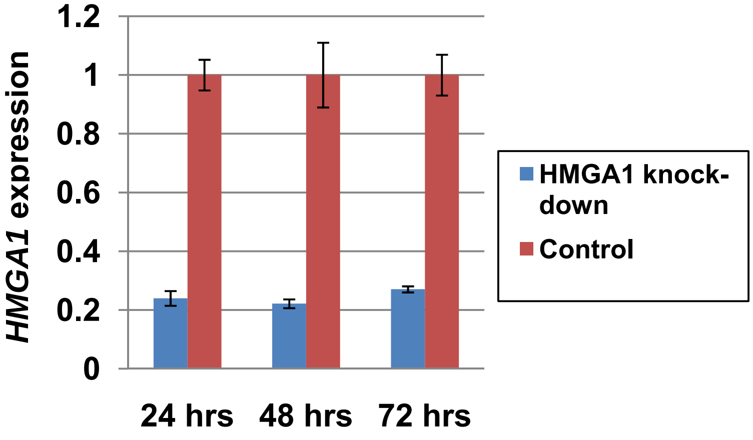

B.)

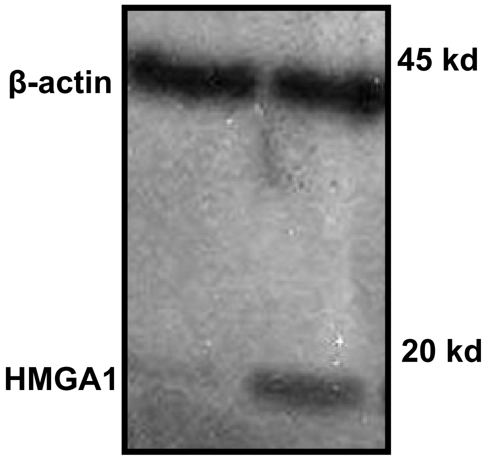

C.)

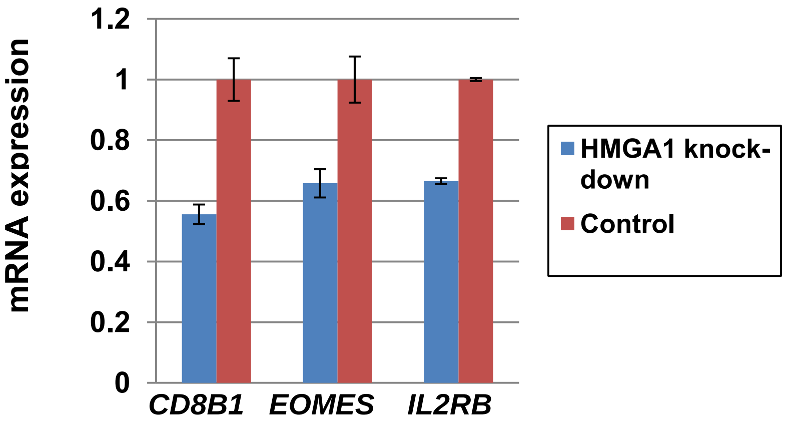

A.) *HMGA1* mRNA expression levels were measured by quantitative (q)RT-PCR in Jurkat cells treated with *HMGA1* siRNA (Dharmacon) compared to controls 24, 48, and 72 hours after transfection. *β-actin* mRNA was used as a loading control. The *HMGA1* mRNA in control cells was assigned a value of 1.0.
B.) Western analysis after treatment with the siRNA for 72 hours. β-actin was used as a loading control for total protein. The HMGA1 protein runs below 20 kd.
C.) Genes that were up-regulated in the *HMGA1* transgenic lymphoid cells were assessed in the human T-ALL cells with or without knock-down of HMGA1 (after 72 hours) by qRT-PCR. We found that 3 genes were significantly repressed in the HMGA1 knock-down cells, including *CD8B1* ($p<0.003$; student's t-test), *EOMES* ($p< 0.0001$; student's t-test), and *IL2RB* ($p<0.0001$; student's t-test).
