# Supplementary material for: HMGA1 drives stem cell, inflammatory pathway, and cell cycle progression genes during lymphoid tumorigenesis
Source: BMC Genomics. 2011 Nov 4;12:549. doi: 10.1186/1471-2164-12-549 (PMC3245506; doi:10.1186/1471-2164-12-549)
Supplement: Additional file 3 — COX-2 data from HMGA1 and control mice. COX-2 mRNA expression in the spleens from HMGA1 or control mice at 2 months is shown. [file 1471-2164-12-549-S3.PDF]

COX-2 mRNA in spleens from _HMGA1_ and control mice at 2 months of age

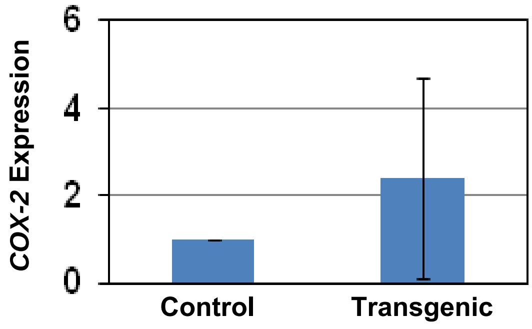

COX-2 mRNA expression levels were assessed using qRT-PCR in lymphoid samples from control and transgenic mice at 2 and 12 months (n = 4 at each time point for each genotype). The bars represent the mean +/- the standard deviation at 2 months. There was elevated *COX-2* expression in ~50% of the transgenics, which accounts for the broad standard deviation shown here.
