# Supplementary material for: HMGA1 drives stem cell, inflammatory pathway, and cell cycle progression genes during lymphoid tumorigenesis
Source: BMC Genomics. 2011 Nov 4;12:549. doi: 10.1186/1471-2164-12-549 (PMC3245506; doi:10.1186/1471-2164-12-549)
Supplement: Additional file 4 — Gene set enrichment analysis. This file includes all overlaps with our gene sets identified by gene set enrichment analysis. [file 1471-2164-12-549-S4.PDF]

Gene set enrichment analysis of gene sets that overlap with the HMGA1 gene sets

**Early in tumorigenesis: Up-regulated genes**		**Early in tumorigenesis: Down-regulated genes**	
Gene set name	p value	Gene set name	p value
IL12PATHWAY	5.87E-05	MOREAUX_TACI_HI_VS_LOW_UP	7.86E-03
GOLDRATH_CYTOLYTIC	6.95E-05		
NKTPATHWAY	9.40E-05		
HSA04060_CYTOKINE_CYTOKINE_RECEPTOR _INTER-ACTION	6.95E-03		
KUMAR_HOXA_DIFF	2.12E-02		
MATSUDA_VALPHAINKT_DIFF	2.33E-02		
BOQUEST_CD31PLUS_VS_CD31MINUS_UP	3.89E-02		
**Established tumors: Up-regulated genes**		**Established tumors: Down-regulated genes**	
Gene set name	p value	Gene set name	p value
LEE_TCELLS3_UP	2.26E-09	AGED_MOUSE_HIPPOCAMPUS_ANY_UP	2.91E-03
STEMCELL_EMBRYONIC_UP	5.53E-09	LAL_KO_3MO_UP	3.16E-03
STEMCELL_NEURAL_UP	7.22E-09	CREB_BRAIN_8WKS_DN	3.29E-03
ZHAN_MM_CD138_PR_VS_REST	1.37E-08	YAGI_AML_PROG_FAB	4.47E-03
LEE_TCELLS2_UP	1.63E-08	BRENTANI_SIGNALING	4.65E-03
GAY_YY1_DN	2.77E-08	HSA04662_B_CELL_RECEPTOR_SIGNALING_PATHWAY	5.50E-03
TARTE_PLASMA_BLASTIC	6.92E-08	LAL_KO_6MO_UP	5.50E-03
VANTVEER_BREAST_OUTCOME_GOOD_VS_POOR_DN	8.92E-08	HSA04810_REGULATION_OF_ACTIN_CYTOSKELETON	5.59E-03
HOFFMANN_BIVSBII_BI_TABLE2	1.51E-07	HSA04664_FC_EPSILON_RI_SIGNALING_PATHWAY	7.43E-03
ADIP_DIFF_CLUSTER5	1.87E-07	INTEGRIN_MEDIATED_CELL_ADHESION_KEGG	1.18E-02
IRITANI_ADPROX_LYMPH	2.10E-07	BASSO_GERMINAL_CENTER_CD40_UP	1.34E-02
BRCA_ER_NEG	2.44E-07	UVC_TTD_4HR_DN	1.37E-02
SERUM_FIBROBLAST_CELLCYCLE	3.69E-07	HADDAD_HPCLYMPHO_ENRICHED	1.49E-02
BRENTANI_CELL_CYCLE	4.11E-07	ET743_SARCOMA_24HRS_DN	1.51E-02
IDX_TSA_UP_CLUSTER3	5.37E-07	VANASSE_BCL2_TARGETS	1.59E-02
BRCA_PROGNOSIS_NEG	7.39E-07	HSA04670_LEUKOCYTE_TRANSENDOTHELIAL_MIGRATION	1.64E-02
CROONQUIST_IL6_RAS_DN	1.37E-06	BRCA_ER_NEG	1.71E-02
HSA04110_CELL_CYCLE	2.25E-06	HSA04650_NATURAL_KILLER_CELL_MEDIATED_CYTOTOXICITY	2.11E-02
HSA04115_P53_SIGNALING_PATHWAY	3.10E-06	UVC_TTD_ALL_DN	2.17E-02
GOLDRATH_CELLCYCLE	4.87E-06	BOQUEST_CD31PLUS_VS_CD31MINUS_UP	2.23E-02

The Molecular Signature Database was interrogated for overlaps with the gene sets identified in our analyses. All overlaps are shown above with the relevant p value.
